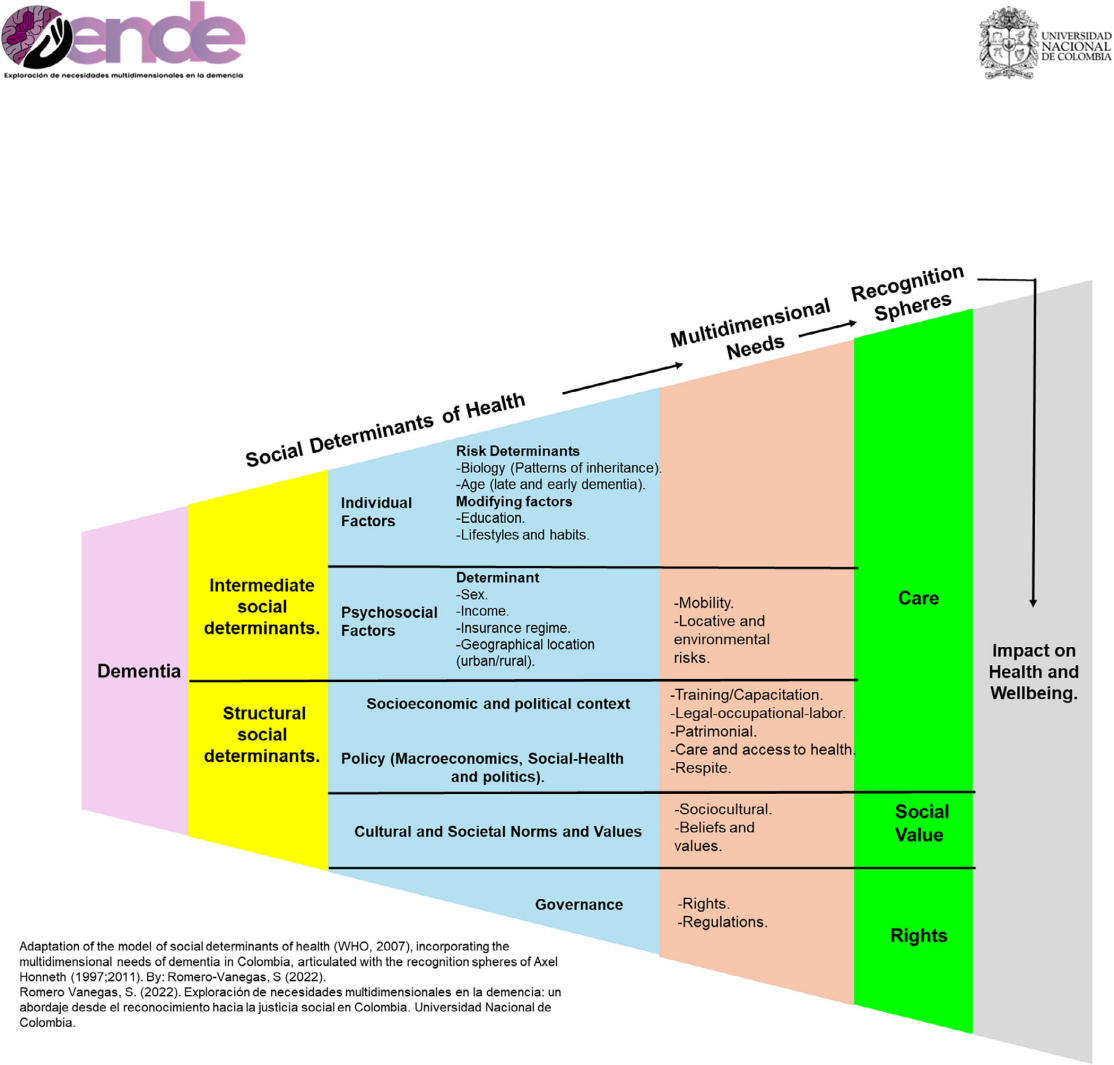# Dementia and the Social Determinants of Health in Colombia

**DOI:** 10.1002/alz.084999

**Published:** 2025-01-09

**Authors:** Sara Julieta Romero‐Vanegas, Rodrigo Pardo, Marisol Moreno‐Angarita

**Affiliations:** ^1^ Universidad Nacional de Colombia, Bogota Colombia

## Abstract

**Background:**

Dementia onset and progression may be related to the social determinants of health (SDH), but information regarding those is scarce. Currently, we still lack knowledge about the role of structural determinants of health in dementia and their relationship to recognition spheres (care, social valuation, needs, and rights). This has been reflected in health policies that do not fulfill the expectations of the patients, caregivers, and health professionals.

**Method:**

We performed an exploratory sequential mixed methods design. We included patients with a mild dementia diagnosis (N:28), caregivers (N: 208), and health professionals (N:39). We used a structured interview, study of cases, and focus groups to gather our information. We performed a triangulation method to evaluate the burden of known and unknown needs and afterwards a theoretical analysis of SDH and recognition theory.

**Result:**

Our results suggest that there are individual factors that become risk determinants for dementia. These factors could be the focus of designing preventive or palliative approaches. Psychosocial variables (place of residence, environmental milieu, mobility facilities, insurance regimen, income level among others) modify the quality of life and well‐being perception.

We found that structural determinants, such as the socioeconomic and political context and politics are related to training, legal‐occupational‐labor, property, care, and access to health and respite needs. Cultural norms and values are associated with sociocultural needs beliefs and values, and governance is linked to rights and regulations. When we associate multidimensional needs with the determinants, we observe that they affect the spheres of recognition generating a negative impact on the disease progress, well‐being, and dignified life.

**Conclusion:**

We found a relationship between SDH and the ten dimensions that we explored through the needs assessment methodology. Unresolved needs and lack of recognition related to social determinants, threaten the way of living and feeling dementia, as well as its evolution and progression. Our result could lead to community empowerment, inclusion, visibility at the decision‐making level, and formulation of policies and healthcare services if we understand the interaction between needs, the social determinants of health and the recognition spheres.